# The “2+1” Simplified Echocardiographic Diagnostic Framework for Constrictive Pericarditis

**DOI:** 10.1016/j.case.2026.02.003

**Published:** 2026-03-24

**Authors:** Aslannif Roslan, Si Ling Soh, Norhaliza Am Haris, Afif Ashari

**Affiliations:** Cardiology Department, National Heart Institute of Malaysia, Kuala Lumpur, Malaysia

## Abstract

• CP can mimic right-sided HF symptoms.
• The 2 + 1 echo framework combines sensitive and specific echocardiographic findings.
• Bedside framework speeds synthesis of complex echocardiographic findings.
• Framework complements, not replaces, guideline imaging and hemodynamics.
• Three CP cases illustrate practical use of the 2 + 1 diagnostic approach.

## Graphical abstract

**Figure:**
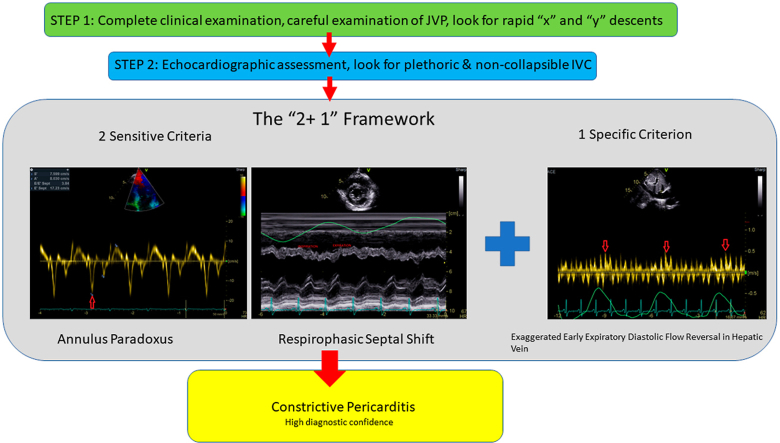

## Introduction

Constrictive pericarditis (CP) should be considered in patients presenting with dyspnea, abdominal bloating, and signs of right-sided heart failure (HF). Clinical assessment, and especially echocardiographic interpretation, can be challenging when the underlying hemodynamic concepts are not well understood. In this case series, we present 3 patients with CP and propose a simple “2 + 1” echocardiographic diagnostic framework that groups established diagnostic findings into 2 sensitive criteria plus 1 more specific criterion. This framework is intended to complement, not replace, guideline-recommended imaging and hemodynamic assessment when indicated. It offers a pragmatic bedside approach to help clinicians rapidly synthesize echocardiographic clues in a busy clinical setting. Its practical value lies in prioritizing the relative importance of key criteria. Similar echocardiographic decision frameworks have been proposed previously, including the practical clinical approach summarized by Miranda *et al.*^1^ Accordingly, we present the 2 + 1 diagnostic framework to be used at the bedside.

## Case Presentations

Case 1 involves a 22-year-old man with no known medical history who presented with a 1-month history of dyspnea and chest discomfort. Examination revealed bilateral pedal edema and ascites with a fluid thrill but normal heart sounds. Careful neck examination showed elevated jugular venous pressure with prominent rapid x and y descents (Video 1). The electrocardiogram and chest radiograph were normal, and laboratory tests, including total white blood cell count, inflammatory markers, high-sensitivity troponin T, and N-terminal pro b-type natriuretic peptide (NT-proBNP), were all within normal limits. The patient had no identifiable risk factors for CP, such as prior cardiac surgery, chemotherapy or radiotherapy, tuberculosis, recurrent pericarditis, or cardiac ablation.

**Figure 1:**
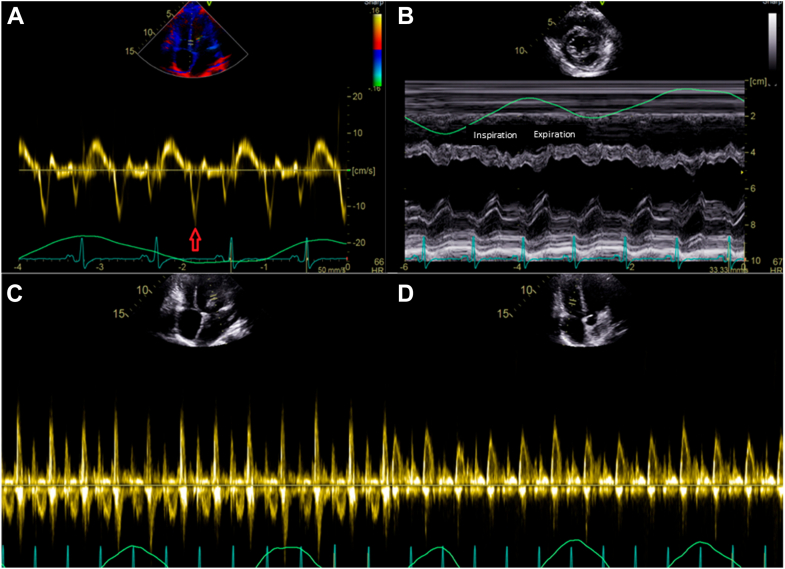
Case 1. Two-dimensional TTE, apical 4-chamber **(A, C, D)** and parasternal short-axis **(B)** views with tissue Doppler **(A)**, M-mode with respirometer **(B)**, and pulsed-wave Doppler of the tricuspid valve **(C)** and mitral valve **(D)**, demonstrates a supranormal septal e’ (*arrow*, 16 cm/s), respirophasic movement of the interventricular septum due to exaggerated interventricular dependence (fulfilling the 2 criteria in the “2 + 1” framework), and a lack of significant respiratory variation across the mitral valve (18%) and tricuspid valve (33%).

**Figure 2:**
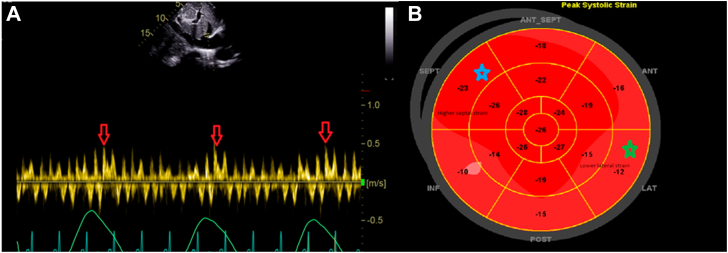
Case 1. Two-dimensional TTE, subcostal view of hepatic vein with respirometer **(A)** and bull's-eye global longitudinal pattern **(B)**, demonstrates exaggerated early expiratory diastolic flow reversal (*red arrows*) and reduction in lateral strain (*green star*) with increased in septal strain (*blue star*).

**Figure 3:**
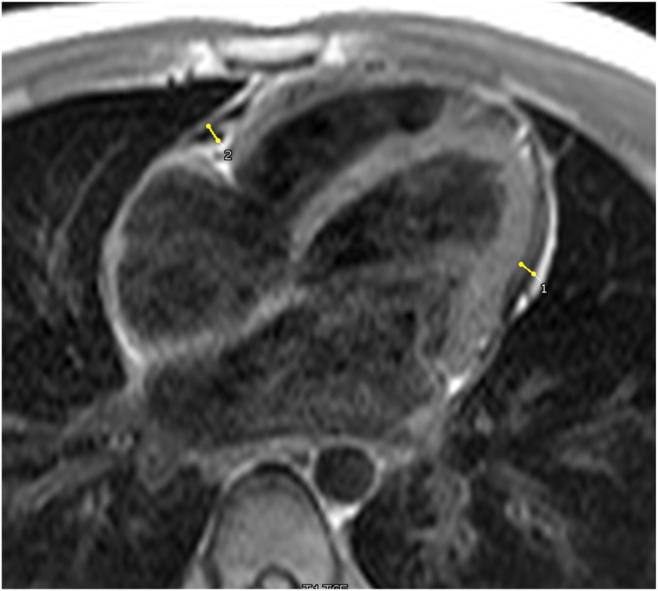
Case 1. Cardiovascular magnetic resonance, T1 turbo spin echo axial image, 4-chamber view, demonstrates pericardial thickening (*arrows*).

Transthoracic echocardiography (TTE) demonstrated normal global left ventricular (LV) systolic function (LV ejection fraction [LVEF] = 63%), an E/A ratio of 3.1, and a plethoric, noncollapsible inferior vena cava (IVC). The study met all components of the proposed 2 + 1 diagnostic framework: annulus paradoxus (paradoxically elevated early diastolic septal e′ velocity in the setting of HF symptoms, in contrast to the low septal e′ expected in restrictive cardiomyopathy [CM]), respirophasic septal shift (leftward septal shift with inspiration and rightward shift with expiration), and the specific finding of exaggerated early expiratory hepatic vein diastolic flow reversal. Annulus reversus, however, was absent, as the septal e′ did not exceed the lateral e′. Respiratory inflow showed 18% mitral and 33% tricuspid inflow variation, which did not meet traditional Doppler cutoffs for CP. Bull's-eye global longitudinal display shows “strain reversus” or “hot septum,” a pattern typical of CP, with higher septal strain and reduced lateral strain, consistent with lateral wall tethering to the pericardium and septal longitudinal compensation (Figures 1 and 2, Video 2).^2^ Cardiovascular magnetic resonance imaging revealed pericardial thickening, a prominent septal bounce, and features of pericardial inflammation where pericardial enhancement was only seen in late gadolinium enhancement images, but negative in short tau inversion recovery-T2 images, consistent with CP (Figure 3, Video 3). Integrating the clinical findings, plethoric noncollapsible IVC, and a positive 2 + 1 echocardiographic framework, a diagnosis of idiopathic CP was made.

Due to social aspects related to financial consideration, the patient was unable to undergo pericardiectomy. The patient initially received a 3-month trial of high-dose aspirin and colchicine; however, the patient experienced no meaningful clinical improvement and will likely require pericardiectomy.

**Figure 4:**
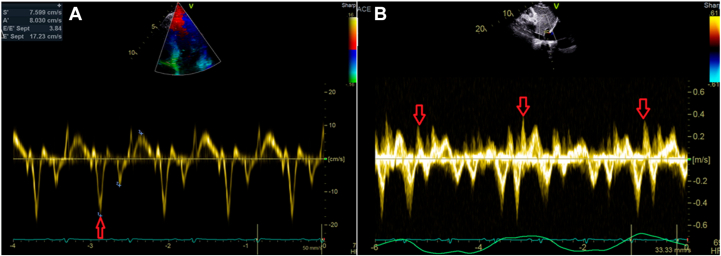
Case 2. Two-dimensional TTE, apical 4-chamber view with tissue Doppler **(A)** and subcostal view of the hepatic vein with respirometer **(B)**, demonstrates annulus paradoxus with supranormal septal e’ at 17.2 cm/s (*single arrow*) and exaggerated early expiratory diastolic flow reversal in the hepatic vein (*3 arrows*).

**Figure 5:**
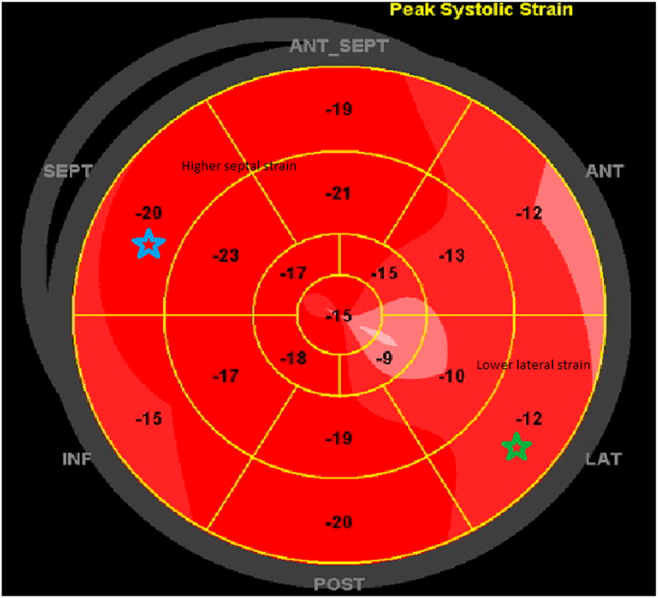
Case 2. Bull's-eyes global longitudinal strain pattern demonstrates typical CP pattern with elevated septal strain (*blue star*) and diminished lateral strain (*green star*).

Case 2 involves a 35-year-old man with a 3-year history of progressive dyspnea associated with abdominal distension and bilateral leg swelling. Physical examination revealed elevated jugular venous pressure, pedal edema, and ascites. The total white blood cell count and high-sensitivity troponin T were normal, while NT-proBNP was mildly elevated at 397 pg/mL. Despite the prolonged symptoms, the patient had consulted 4 different cardiologists over 3 years without receiving a definitive diagnosis and had no known predisposing risk factors for CP. Transthoracic echocardiography demonstrated normal global LV systolic function (LVEF = 55%), an elevated E/A ratio of 3.0, and a plethoric, noncollapsible IVC. The study fulfilled our 2 + 1 diagnostic framework, showing annulus paradoxus, respirophasic septal shift, and exaggerated early expiratory diastolic flow reversal in the hepatic vein (Figure 4; Video 4). Additional supportive findings included annulus reversus, significant mitral inflow variation of 31% (with nonsignificant tricuspid inflow variation at 31%), beat-to-beat septal bounce, a bull's-eye global longitudinal strain pattern typical of CP (Figure 5), and evidence of pericardial thickening (Video 5). Taken together, the characteristic clinical presentation, plethoric noncollapsible IVC, and a positive 2 + 1 diagnostic framework supported a diagnosis of idiopathic CP.

**Figure 6:**
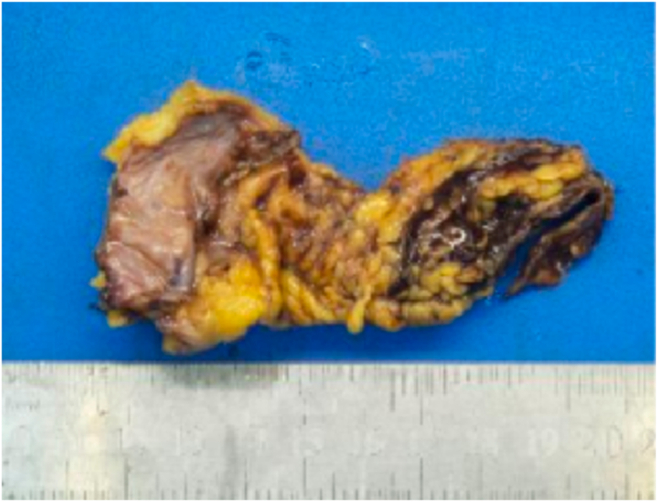
Case 2. Gross pathologic specimen demonstrates that the pericardium is thickened with hyalinized fibrous tissue consistent with CP.

After 3 years of unrelenting symptoms, the patient underwent total pericardiectomy. Four months postoperatively, the patient had fully recovered, with complete resolution of dyspnea and pedal edema and normalization of jugular venous pressure and NT-proBNP. Histopathologic examination of the surgical specimen confirmed a thickened, fibrotic pericardium, corroborating the diagnosis of CP (Figure 6).

**Figure 7:**
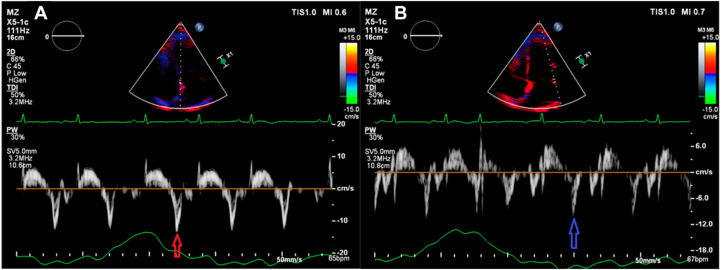
Case 3. Two-dimensional TTE, apical 4-chamber view **(A, B)** showing very high (supranormal) septal e’ (*red arrow*); annulus paradoxus and lower lateral e’ (*blue arrow*) vs higher septal e’; and annulus reversus.

**Figure 8:**
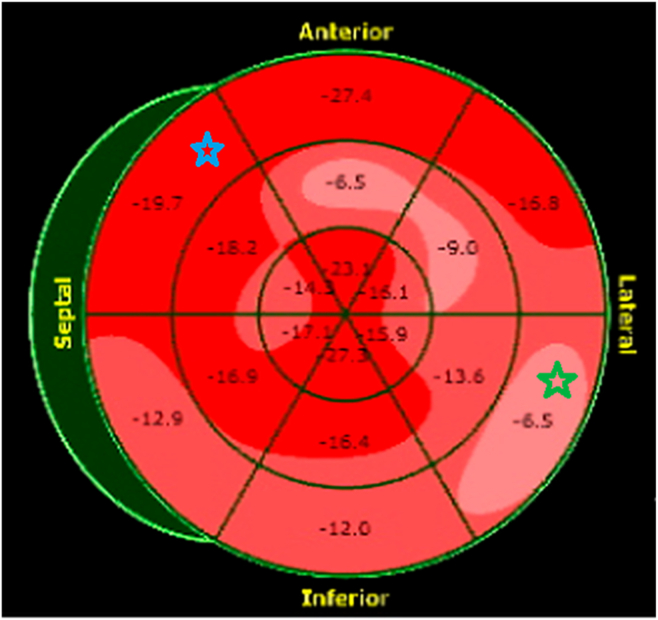
Case 3. Bull's-eye global longitudinal strain demonstrates a similar pattern with the previous 2 patients where septal strain (*blue star*) is higher than lateral strain (*green star*).

**Figure 9:**
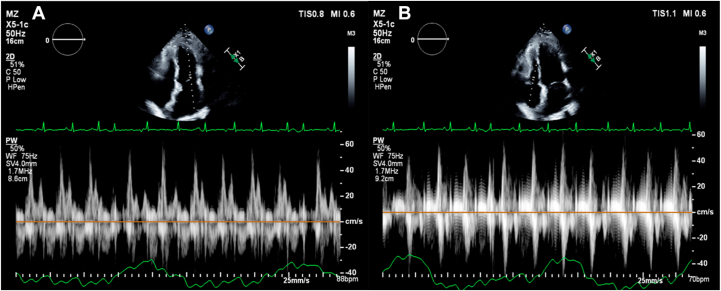
Case 3. Two-dimensional TTE, apical 4-chamber view **(A, B)** demonstrates mitral and tricuspid inflow variations (both 24%) that did not meet the traditional CP criteria.

**Figure 10:**
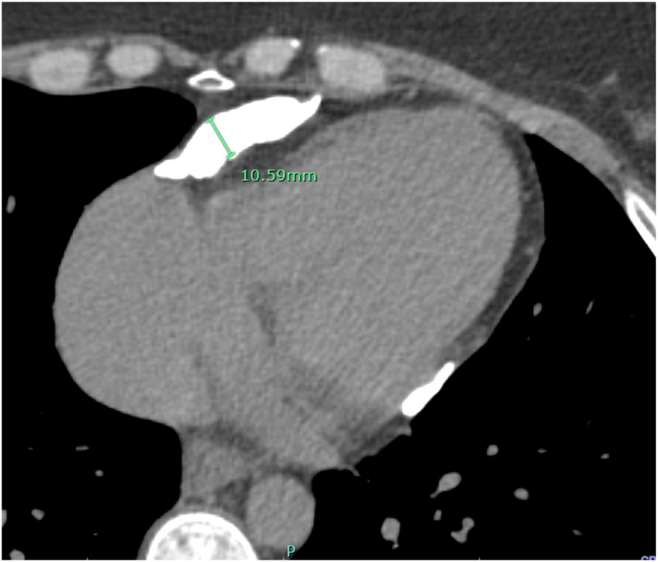
Case 3. Chest computed tomography demonstrates significant pericardial calcification, especially at the lateral wall of the pericardium up to 10.6 mm (*green line*).

Case 3 involves a 52-year-old woman who presented with a 5-month history of exertional dyspnea, abdominal distension, and bilateral leg swelling. Neck examination revealed elevated jugular venous pressure with rapid x and y descents (Video 6). The patient had a history of recovered peripartum CM but no other recognized risk factors for CP. The total white blood cell count, high-sensitivity troponin T, and NT-proBNP were all within normal limits. Transthoracic echocardiography showed normal global LV systolic function (LVEF = 55%) with an E/A ratio of 1.5. The study fulfilled our 2 + 1 diagnostic framework and additionally demonstrated annulus reversus (Figure 7). An ultrasound enhancement agent was used to better delineate the respirophasic septal motion (Video 7). Global longitudinal strain analysis revealed the characteristic bull's-eye pattern of CP, with relatively higher septal strain and reduced lateral strain (Figure 8). Although mitral and tricuspid inflow variations (24% and 24%, respectively; Figure 9) did not meet traditional Doppler thresholds for CP, the combination of the clinical presentation, a plethoric, noncollapsible IVC, and a positive 2 + 1 echocardiographic profile strongly supported the diagnosis. Cardiac computed tomography further demonstrated diffuse pericardial thickening and calcification consistent with CP (Figure 10), and a diagnosis of CP was established.

**Figure 11:**
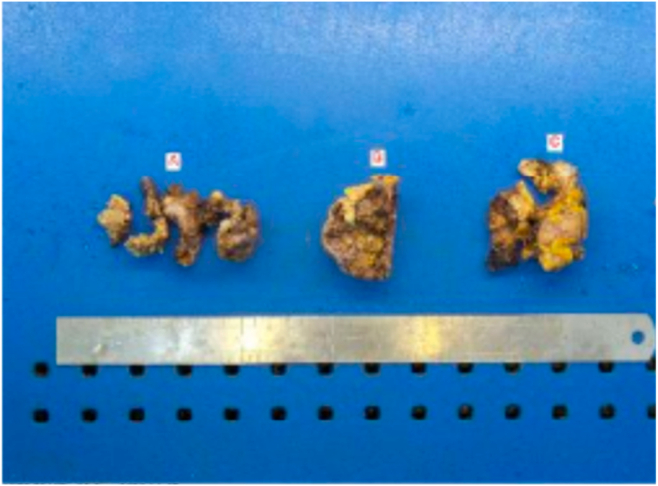
Case 3. Gross examination of pericardial specimens A, B, and C (representing different portions excised by the surgeon) demonstrates diffuse pericardial thickening with hyaline degenerative change, focal calcification, and viable thickened fibro collagenous tissue.

The patient subsequently underwent total pericardiectomy. Histopathologic examination of the surgical specimen confirmed a thickened, fibrotic pericardium consistent with CP (Figure 11). A follow-up telehealth visit confirmed that the patient was doing well postoperatively with complete resolution of symptoms.

## Discussion

Constrictive pericarditis was first described by Chevers in 1842.^3^ For many years, right heart catheterization served as the primary diagnostic modality, based on characteristic hemodynamic findings, including elevated and equalized biventricular end-diastolic pressures, elevated right atrial pressure, and the classic “square root sign” on ventricular pressure tracings.^4^ Today, however, echocardiography has become the first-line imaging modality for diagnosing CP. This shift is important because, although right heart catheterization has long been considered the reference standard, many of its traditional criteria show substantial overlap between CP and restrictive CM.^5^ As a result, patients with CP are frequently misdiagnosed, in part because they often present with HF symptoms, despite normal LV systolic function, and in part because limited familiarity with key echocardiographic concepts can lead to overly complex or inaccurate interpretation.

This case series aims to simplify echocardiographic interpretation by introducing the 2 + 1 diagnostic framework, which combines 2 highly sensitive parameters with 1 highly specific parameter, derived from established studies on CP.^6^ In 2 of the 3 cases, the diagnosis was ultimately confirmed by surgical findings at pericardiectomy. The principal differential diagnosis of CP is restrictive CM. Both conditions classically have normal (nondilated) LV size and normal or near-normal LV systolic function, especially early in the disease course. That's why we can't reliably distinguish them from LV size and function alone. However, in restrictive CM, LV systolic function may be normal early and more likely to decline because myocardium is intrinsically diseased. Notably, the echocardiographic criteria in this diagnostic framework are not seen in restrictive CM: Septal e′ is elevated in CP but reduced in restrictive CM; respirophasic septal shift is absent in restrictive CM; and hepatic vein Doppler in CP shows exaggerated expiratory diastolic flow reversal, in contrast to the exaggerated inspiratory diastolic flow reversal typical of restrictive CM. In addition, supportive findings such as beat-to-beat septal bounce are absent in restrictive CM, and global longitudinal strain in restrictive CM does not demonstrate the characteristic patterns observed in these 3 CP cases.

The 2 sensitive parameters are annulus paradoxus (sensitivity 83%), defined as a supranormal septal e′ velocity (≥9 cm/s) in a patient with HF symptoms, and respirophasic septal shift due to increased interventricular dependence (sensitivity 93%), in which the septum moves leftward during inspiration and rightward during expiration. The specific parameter (specificity 91%) is exaggerated early expiratory diastolic flow reversal in the hepatic vein, which occurs when rightward septal shift during expiration increases right-sided pressures and transmits this back pressure to the hepatic veins.^7^

Additional supportive but nonmandatory criteria include annulus reversus (due to tethering of the lateral LV wall), mitral inflow variation >25%, and tricuspid inflow variation >40% (with some variability in reported cutoffs across studies), beat-to-beat septal bounce (often mistaken for respirophasic septal shift), and global longitudinal strain demonstrating relatively normal septal compared with lateral strain.^2^^,^^7^ Importantly, the absence of pericardial thickening on echocardiography or computed tomography does not exclude CP, and its presence, while supportive, is not pathognomonic.^8^ Current guidelines and consensus documents emphasize that these thresholds should be interpreted in clinical context rather than as rigid binary cutoffs. The proposed 2 + 1 diagnostic framework is a pragmatic approach derived from the reported sensitivity and specificity of individual criteria, combined with clinical experience, to streamline the echocardiographic diagnosis of CP; it is not intended to replace guideline-based diagnostic pathways. Future prospective studies in larger cohorts are necessary to validate this diagnostic framework. This approach remains aligned with existing guideline recommendations for pericardial disease, which endorse echocardiography as the first-line imaging modality and emphasize the complementary role of multimodality imaging and invasive hemodynamic assessment in selected cases.

## Limitations

This report describes only 3 patients from a single center, and the proposed 2 + 1 echocardiographic diagnostic framework has not been formally validated. Our findings are therefore hypothesis-generating and should not be extrapolated to all patients with suspected CP. The generalizability of the 2 + 1 diagnostic framework may be limited by echocardiographic image quality, operator experience, respiratory effort, and the presence of coexisting cardiac or pulmonary disease. We did not perform invasive hemodynamic assessment in all cases, which may be viewed as a limitation; however, in each patient, the final diagnosis was supported by multimodality imaging and/or surgical findings. Prospective studies in larger cohorts are necessary to validate and to determine the sensitivity, specificity, and reproducibility of this diagnostic framework and to clarify how it might best complement guideline-based diagnostic algorithms.

## Conclusion

In this case series, we illustrate how a pragmatic 2 + 1 echocardiographic diagnostic framework can help clinicians recognize CP by prioritizing 2 sensitive findings together with 1 more specific criterion. Across 3 presentations of right-sided HF physiology, synthesizing these key features at the bedside supported timely diagnostic reasoning and reinforced the hemodynamic concepts that underlie CP. Used as a complement to guideline-recommended imaging and, when needed, invasive assessment, this simplified approach may reduce cognitive overload and improve consistency in echocardiographic interpretation in busy clinical practice.

## Ethics Statement

The authors declare that the work described has been carried out in accordance with The Code of Ethics of the World Medical Association (Declaration of Helsinki) for experiments involving humans

## Consent Statement

Complete written informed consent was obtained from the patient (or appropriate parent, guardian, or power of attorney) for the publication of this study and accompanying images.

## Funding

The authors declare that this report did not receive any specific grant from funding agencies in the public, commercial, or not-for-profit sectors.

## Disclosure Statement

The authors reported no actual or potential conflicts of interest relative to this document.

## Supplementary Data

Video 1 Case 1. Neck examination of the jugular venous pulse demonstrates an elevated jugular venous pressure with rapid x and y descents.

Video 2 Case 1. Two-dimensional TTE, apical 4-chamber zoom view, demonstrates respirophasic septal shift, moving to the left with inspiration and right on expiration with beat-to-beat “septal bounce.”

Video 3 Case 1. Cardiovascular magnetic resonance, free breathing cine 4-chamber view, demonstrates respirophasic septal shift and septal bounce consistent with CP.

Video 4 Case 2. Two-dimensional TTE, short-axis view at the mitral valve level, demonstrates respirophasic septal shift and beat-to-beat septal bounce.

Video 5 Case 2. Two-dimensional TTE, subcostal 4-chamber view, demonstrates thickened pericardium at the lateral wall of the right ventricle.

Video 6 Case 3. Neck examination of the jugular venous pulse demonstrates an elevated jugular venous pressure with rapid x and y descents typical of CP.

Video 7 Case 3. Two-dimensional TTE, apical 4-chamber zoom view after the intravenous administration of an ultrasound enhancing agent, demonstrates normal LV size and function with respirophasic septal shift and septal bounce.
